# Development of a Duck Genomic Reference Material by Digital PCR Platforms for the Detection of Meat Adulteration

**DOI:** 10.3390/foods10081890

**Published:** 2021-08-15

**Authors:** Xiaoyun Chen, Yi Ji, Kai Li, Xiaofu Wang, Cheng Peng, Xiaoli Xu, Xinwu Pei, Junfeng Xu, Liang Li

**Affiliations:** 1 State Key Laboratory for Managing Biotic and Chemical Threats to the Quality and Safety of Agro-Products, Zhejiang Academy of Agricultural Sciences, Hangzhou 310021, China; xiaoyunchen_2016@163.com (X.C.); jymemory12138@163.com (Y.J.); yywxf1981@163.com (X.W.); pc_phm@163.com (C.P.); xuxiaoli@zju.edu.cn (X.X.); 2 Biotechnology Research Institute, Chinese Academy of Agricultural Sciences, Beijing 100081, China; likaij@163.com (K.L.); peixinwu@caas.cn (X.P.)

**Keywords:** certified reference material, digital PCR, duck *interleukin 2*, mitochondrial gene, meat adulteration

## Abstract

Low-cost meat, such as duck, is frequently used to adulterate more expensive foods like lamb or beef in many countries. However, the lack of DNA-based reference materials has limited the quality control and detection of adulterants. Here, we report the development and validation of duck genomic DNA certified reference materials (CRMs) through the detection of the duck *interleukin 2* (*IL2*) gene by digital PCR (dPCR) for the identification of duck meat in food products. The certified value of *IL2* in CRMs was 5.78 ± 0.51 × 10^3^ copies/μL with extended uncertainty (coverage factor *k* = 2) based on *IL2* quantification by eight independent collaborating laboratories. Quantification of the mitochondrial gene *cytb* revealed a concentration of 2.0 × 10^6^ copies/μL, as an information value. The CRMs were also used to determine the limit of detection (LOD) for six commercial testing kits, which confirmed that these kits meet or exceed their claimed sensitivity and are reliable for duck detection.

## 1. Introduction

Meat adulteration has become a major global issue. Fraud by substitution or adulteration with inexpensive raw materials poses an attractive shortcut for unscrupulous food producers, although it is fraught with potential public health risks [1]. In order to definitely show that a product has been adulterated or fraudulently labeled, the product composition must be first determined for comparison of its authenticity with the description provided on its label. This process requires quantitative analysis of characteristic compounds or analytes specific to the ingredient in question, or other evidence that it is present in concentrations at or above levels required by regulatory agencies [2]. Therefore, the development of an accurate and reliable method for determining the content of specific meat components in food products has major economic implications and far-reaching social significance.

Traditional methods for species identification have historically relied on anatomy, histology, sensory judgment, chemistry, electrophoresis, chromatography, and immunology [3]. However, these approaches are each accompanied by limitations in their accuracy and/or sensitivity. To address this issue, recent studies have developed polymerase chain reaction (PCR)-based assays to accommodate the discriminatory capability necessary for identification of specific ingredients or contaminants. The most widely used methods are DNA-based screens [4], including gel-based PCR [5], real-time qPCR [6], multiplex PCR [7], digital PCR [8], isothermal nucleic acid amplification [9,10,11], and other PCR techniques [12,13]. The recent and extremely rapid expansion in detection methods and the standardization of detection methods for animal-based food products has inadvertently circumvented some steps that are essential to ensure the rigor and quality of data, which is compounded by a lack of reference materials. These factors together can incur a bottle-neck in data quality assurance. To rectify this issue in data quality assurance in food production, many reference materials are urgently needed for the ongoing evaluation of the methods used for quality control.

A reference material (RM) is sufficiently homogeneous and stable with respect to one or more specified properties that have been established to be fit for its intended use in a measurement process. Certified reference materials (CRMs) are reference materials (RMs) characterized using a metrologically valid procedure for one or more specified properties, accompanied by an RM certificate [14], which can be used for calibration of a measurement system, assessment of a measurement procedure, for assigning values to other materials, and quality control. Several DNA-based CRMs have been developed, such as for genetically modified organisms [15,16,17], cancer diagnosis [18,19,20], foodborne pathogens [21], and forensic science [22,23].

Due to the increasingly high frequency of adulteration of meat products with inexpensive duck, this work aimed to develop a novel DNA reference material for duck meat through the targeted detection and quantification of the duck *IL2* gene. Here, we describe the preparation, homogeneity, and short- and long-term stability of the gDNA materials. The mean *IL2* copy number values of these CRMs for duck were validated through digital PCR (dPCR) by eight independent collaborating diagnostic laboratories, and the materials were certified by China’s State Administration for Market Regulation. In addition, the reference materials were used to evaluate the limit of detection for six commercial testing kits. These CRMs are intended for qualitative and quantitative screening for duck meat in meat products through detection of the *IL2* gene. These CRMs can also be used for validation of other quantitative DNA-based methods and laboratory quality control.

## 2. Materials and Methods

### 2.1. Extraction and Evaluation of Duck Genomic DNA

Duck leg meat was provided by the Institute of Animal Husbandry and Veterinary Science, Zhejiang Academy of Agricultural Sciences. Duck leg meat was used, and a sterile scalpel was used to remove the skin and cut it into small pieces, after which it was blended in Philips Mixture HR2027 (Hongkong, China), and then transferred to a Nalgene^®^ 3118-0050 Oak Ridge Centrifuge Tube. Genomic DNA extraction was performed using a Simgen Animal Tissue DNA Midi Kit (Hangzhou, China). The integrity of the DNA critically affects the success of the characterization, which was analyzed by gel electrophoresis using a DYY-6C gel electrophoresis system (Liuyi Biotechnology, Beijing, China). The quality and purity of gDNA was evaluated at 230 nm, 260 nm, and 280 nm by the UV absorbance method (Nanodrop^TM^ 2000, Thermo Fisher, Wilmington, DE, USA). The extracted gDNA was quantified using the Quant-iT™ dsDNA PicoGreen^®^ Kits (Invitrogen, Shanghai, China) according to the manufacturer’s instructions using a lambda DNA standard solution.

### 2.2. Digital PCR Assay

#### 2.2.1. Certified Value

**Figure 1:**
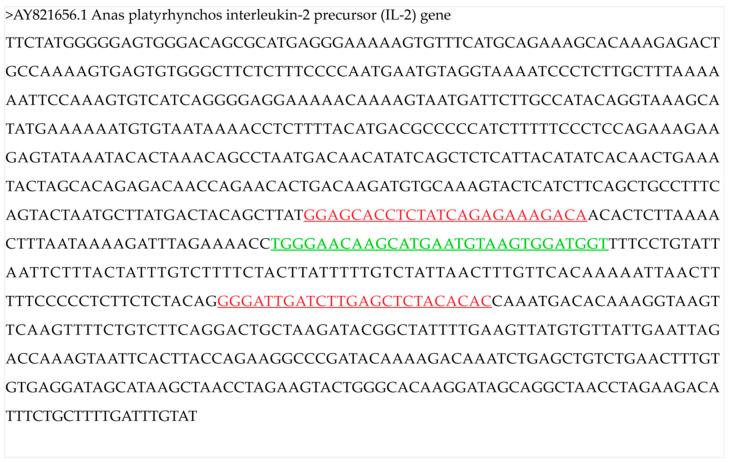
Sequence of the *interleukin* *2* precursor gene (AY821656.1). The primers (red) and probe (green) for the digital PCR are underlined. The amplicon size was 212 bp.

The target gene is a single-copy nuclear gene in the genome. The primers and probe (Figure 1) used for specific quantification were as described previously [24]. The primers and probe were synthesized by Sangon (Shanghai, China). The amplicon size was 212 bp. The primer and probe sequences are shown as follows:

5′-GGAGCACCTCTATCAGAGAAAGACA-3′;
5′-GTGTGTAGAGCTCAAGATCAATCCC-3′;
5′-FAM-TGGGAACAAGCATGAATGTAAGTGGATGGT-BHQ1-3′.

Optimization of the PCR condition is critical for developing a PCR-based method for the specific detection of duck. This study fully optimized the key parameters of real-time qPCR and digital PCR, including specificity, oligonucleotide concentration, annealing temperature, dynamic range, the limit of detection, and the limit of quantification (data not shown). The optimized PCR reaction contains 10 µL of 2X ddPCR Supermix for probes (no dUTP) (Bio-Rad, Hercules, CA, USA), 1 µL of each primer (0.5 µM), 0.5 µL of probe (0.25 µM), 2 µL of DNA template, and 5.5 µL of ddH2O. After that, the 20 µL of the reaction mixture was then loaded on eight-channel disposable droplet generator cartridges (Bio-Rad). Droplets were generated with 70 µL of droplet generation oil (Bio-Rad) in the droplet generator of the QX200 system (Bio-Rad). The generated droplets were transferred to a 96-well PCR plate (Bio-Rad). The amplification was carried out at a uniform ramp rate of 2.5 °C/s for 95 °C for 10 min, 45 cycles of 95 °C for 15 s followed by 60 °C for 1 min, and a final enzyme deactivation at 98 °C for 10 min. Fluorescent signals from amplified droplets were captured individually in the QX200 droplet reader (Bio-Rad) and analyzed with QuantaSoft 1.6.6.0320 software. The target concentrations were reported as the number of copies per µL of PCR reaction after correction with the Poisson distribution.

#### 2.2.2. Information Value

A mitochondrial *cytb* gene TaqMan MGB probe assay [25] was used. For absolute quantification, the primers and probe were fully performed in a QX200 ddPCR system. Primers and probes were purified with high-performance liquid chromatography. The preparation of the reaction mixture and optimized PCR thermal procedure is listed in the Supplementary Materials. The primers and probes are as follows:

5′-GGCCACACAAATCCTCACAG-3′;
5′-TGTGTTGGCTACTGAGGAGAAA-3′;
5′-FAM- CCTACTGGCTATGCACTACACCGCAGAC-BHQ1-3′.

### 2.3. Homogeneity Testing

Most CRMs are prepared as batches of bottles or vials. It is important that all units are the same within the stated uncertainty for each property value. ISO 17034 accordingly requires the assessment of the homogeneity of a certified reference material. Homogeneity includes within-unit homogeneity and between-unit homogeneity. It is always necessary to assess the between-unit homogeneity, which was assessed to ensure the equivalence between different units following the Section 7.5.2 of the ISO Guide 35:2017. Furthermore, the within-unit homogeneity is directly reflected in the minimum size of the subsample. The homogeneity of the *IL2* duck gene was evaluated by randomly selecting 15 units from the CRM candidates. Three subsamples from different positions of each unit were taken and measured by dPCR. Measurement results were used for the assessment of homogeneity. Statistical analysis of the data was performed for a 95% confidence level. The values of the copy number concentration for this *IL2* gene of the duck samples were analyzed using the F test and compared with the significance of the calculated F value of the copy number concentration and the critical F_0.05(14, 30)_ value of 2.04.

An experimental assessment must consist of a determination of the minimum sample intake. It is always essential to ensure that the sample intake is sufficient. The between-unit study does not provide such assurance, so the experimental within-unit homogeneity study should be carried out.

### 2.4. Stability Monitoring

Stability is an important parameter for the reference material. The stability of all CRMs should be assessed. Two types of stability are relevant in the production of reference material: the long-term stability and transportation stability (short-term stability). This CRM is the DNA solution, so it needs to be repeatedly frozen and thawed, and thus the freeze-thaw stability is a concern. Stability testing was carried out to evaluate the influence of different storage temperatures and times on the CRMs using digital PCR (dPCR). The uncertainty of stability needs to include the uncertainty of long-term stability and short-term stability.

Transportation stability (short-term stability) is a property of the material referring to stability under expected transport conditions. Extreme high temperatures cannot be ruled out during transportation, so the CRM units were stored at 4 °C, 25 °C, 37 °C, and 60 °C for 1, 3, 5, 7, and 14 days. The samples were stored at −70 °C after sampling. Three tubes were randomly selected for each storage temperature and each tube was sampled 3 times. The digital PCR tested the stability of *IL2* gene of the duck samples. The *t*-test was performed on the chosen dates to evaluate the short-term stability of the CRMs. The *t*-test results showed no significant slope (β1) for the CRMs at 4 °C, 25 °C, and 37 °C at the 95% confidence level during the two weeks.

Long-term stability studies are conducted to assess stability under storage conditions specified for the lifetime of the product. The long-term stability study was extended to 6 months, the *IL2* gene of the duck CRMs was evaluated by analyzing 3 tubes stored at −20 °C for 1, 2, 4, and 6 months. The samples were stored at −70 °C after sampling. Three tubes were randomly selected for each storage temperature and each tube was sampled 3 times by the digital PCR. Because the *IL2* gene of the duck CRMs is the DNA solution, their freeze-thaw stability needs to be implemented. This batch of CRMs was 100 μL per tube and the minimum sample was 2 μL. In the experiment, 3 tubes of the *IL2* gene of the duck CRMs were taken, repeated freezing-thawing was performed 10 times, and samples were taken 3 times per tube by the digital PCR.

### 2.5. Collaborative Characterization

The property value of this batch of CRMs was determined by eight laboratories (Supplementary Table S1) using dPCR. The eight laboratories were engaged in DNA measurement for a long time and have a certain technical authority.

The gDNA samples of the *IL2* gene of ducks and the related ddPCR reagents were mailed to each participating laboratory in a closed box filled with dry ice. Each laboratory received two gDNA samples (label: duck1, duck2), each with a volume of 100 μL. In order to simplify the sampling procedure and reduce the deviation, primers/probes were mixed according to the proportion before sending them to each laboratory. The participating laboratories were requested to measure the copy number concentration by the operation protocol of dPCR experiments. Each sample was repeatedly measured four times, and a total of eight subsamples were required simultaneously on the same PCR plate for each participant. The eight participants need to export data and original files to the organization lab. The returned data were analyzed according to the requirements of ISO Guide 35:2017.

### 2.6. Evaluation of Limit of Detection of Commercial Assay Kits

In this experiment, six commercial duck-derived detection qPCR kits were purchased. There were four mitochondrial gene targets and two nuclear gene kits. Company and product information are summarized in Supplementary Table S2. The CRM was serially diluted and applied to limit of detection (LOD) probit regression analysis for six diagnostic assays [26]. Each of the concentration levels were tested with multiple replicates per concentration according to the manufacturer’s instructions with blank controls. Probit regression analysis of 95% hit rates was performed with SPSS 16.0 software (SPSS Inc., Chicago, IL, USA).

## 3. Results

### 3.1. gDNA Extracted from Commercially Obtained Duck Leg Meat

Prior to testing for adulteration, we first sought to confirm the quality of gDNA extracted from leg meat samples of duck obtained from the Institute of Animal Husbandry and Veterinary Science, Zhejiang Academy of Agricultural Sciences. To this end, we first analyzed the extracted gDNA by 1% agarose gel electrophoresis and found neither a visible smear nor an RNA band, which indicated that the gDNA was intact (i.e., not degraded) and that RNA had been removed during the isolation process. In addition, the quantity and quality of each gDNA extraction were confirmed in six technical replicates using a Nanodrop 2000 spectrometer (Thermo Scientific, Wilmington, DE, USA). The A260/A280 ratio averaged 1.98, while the average A260/A230 ratio was 2.28, indicating high purity. The quantification of gDNA concentration by the Qubit 2.0 Fluorometer (Invitrogen) showed that the samples contained approximately 100 ng/μL. DNA solutions were then diluted to concentrations of 6.5 ng/μL with 0.1 × Tris-EDTA (TE) buffer and stored in a −20 °C freezer.

### 3.2. Bottling and Storage/Homogeneity Testing and Minimum Sample Intake

In order to establish protocols for future sample collection and preparation, we next established standardized steps for the proper storage and handling of DNA extractions following methods described before [17]. The gDNA solutions were sterilized using a 0.2 µm nylon filter and 100 µL aliquots were placed in low static, sterilized polypropylene microcentrifuge tubes, with approximately 5 × 10^3^ copies of duck gDNA per tube. Cardboard freezer boxes accommodating 100 samples each were used for storage, and random samples were taken from each box for testing homogeneity as well as short- and long-term stability. All samples were stored in 4 °C refrigerators in the dark.

In order to establish the protocol for evaluating the duck certified reference material (CRM), we examined the homogeneity of the *IL2* gene in our gDNA extractions using a random stratified sampling method [27] to select 15 units from among 500 CRM candidate samples. Three subsamples were taken from different vial positions for each sample and analyzed by digital PCR (dPCR) to determine whether this technique was sufficiently sensitive to identify differences in *IL2* uniformity within and between samples (for raw data see Supplementary Table S3). Significant differences in the *IL2* copy number among within-vial subsamples and between different samples were then determined using an F test to compare the F_calculated_ with the F_critical_ value (2.04) for a 95% confidence level. The results showed that the F_calculated_ (1.53) value was lower than the F_critical_ (2.04) value (Table 1), which thus indicated high homogeneity among the CRM gDNA samples in this batch.

### 3.3. Stability Monitoring and Freeze-Thaw Cycles Testing

The stability of the properties of interest (in this case, the duck *IL2* copy number) represents an essential feature of any given reference material. It is well-known that long-term stability is related to storage conditions, while short-term stability is related to external factors during sample transportation. Moreover, stability studies can be categorized as either classical stability or synchronous stability studies. In this work, we incorporated data from other international studies [16,19,28] characterizing DNA standard materials in a classical stability assessment of duck *IL2* gene stability at −70 °C. Under these storage conditions, we found that the *IL2* copy number of the candidate duck CRMs did not change. It warrants mention here that the copy number stability at other storage temperatures requires a synchronous stability study for comparison with storage at −70 °C.

**Figure 2:**
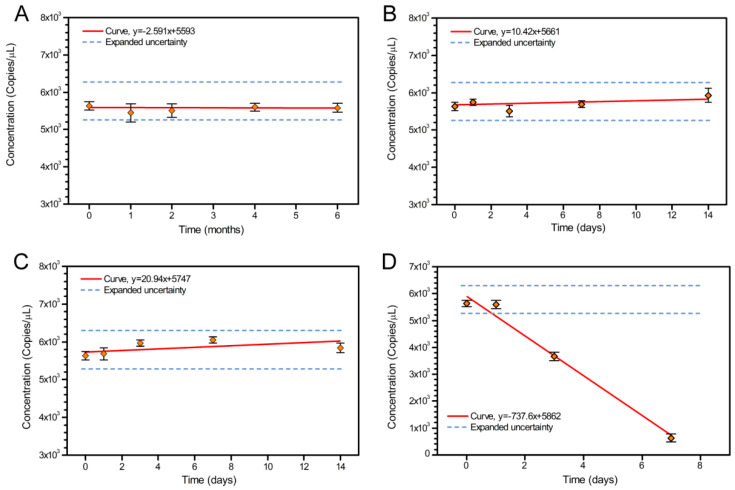
Trends and concentration of stability analysis for the candidate materials: (**A**) long–term stability at −20 °C and (**B**–**D**) short–term stability at 4 °C, 25 °C, and 60 °C. The error bars represent the standard deviation of within–bottle triplicate subsamples.

Transportation stability (short-term stability) is a property of the material that describes its stability under expected transport conditions in order to guide proper handling methods prior to evaluation. Since extremely high temperatures cannot be ruled out during transportation, gDNA CRMs were stored at 4 °C, 25 °C, and 60 °C for 1, 3, 5, 7, and 14 days. Three tubes were randomly selected for storage at each temperature, and each tube was tested in three technical replicates by digital PCR. The results of the *t*-test showed no significant differences in the slope (β1) between the CRMs stored for two weeks at 4 °C or 25 °C at a 95% confidence level (Supplementary Table S4 and Figure 2). However, at 60 °C, the *IL2* copy number significantly decreased after a single day of storage. The relative uncertainty in the *IL2* copy number due to instability at 25 °C storage was calculated to be 0.035. Collectively, these results of short-term CRM stability analysis indicated that duck gDNA is stable under room temperature (25 °C) storage and transport for up to 14 days, though we advise cold chain transportation to minimize the likelihood of degradation.

In order to determine the long-term stability of the CRMs, three randomly selected tubes were stored at −20 °C and tested for the *IL2* copy number by dPCR at 1, 2, 4, and 6 months time points. The results of this analysis showed no significant differences in the *IL2* copy number among any of the time points throughout the 6-month experiment at −20 °C (*p* < 0.05) (Supplementary Table S5 and Figure 2). We then estimated a 0.020 relative uncertainty of the copy number instability caused by long-term storage at −20 °C. Collectively, these results showed that gDNA CRMs could be stably stored at −20 °C for at least 6 months.

Finally, we investigated whether freeze-thaw cycles could adversely affect the *IL2* copy number. Since this batch of CRM samples each contained 100 μL per tube and our above results showed that 2 μL of template was appropriate for analysis, we then randomly selected three tubes of duck CRM gDNA and performed 10 repeated freezing-thaw cycles. Subsequently, dPCR was performed using each sample in three technical replicates, which showed no significant difference in the duck *IL2* gene copy number (*p* > 0.05) among samples. These results indicated that duck gDNA CRMs are stable and robust against damage due to repeated freezing and thawing (data not shown).

### 3.4. Characterization of Certified and Information Value

**Figure 3:**
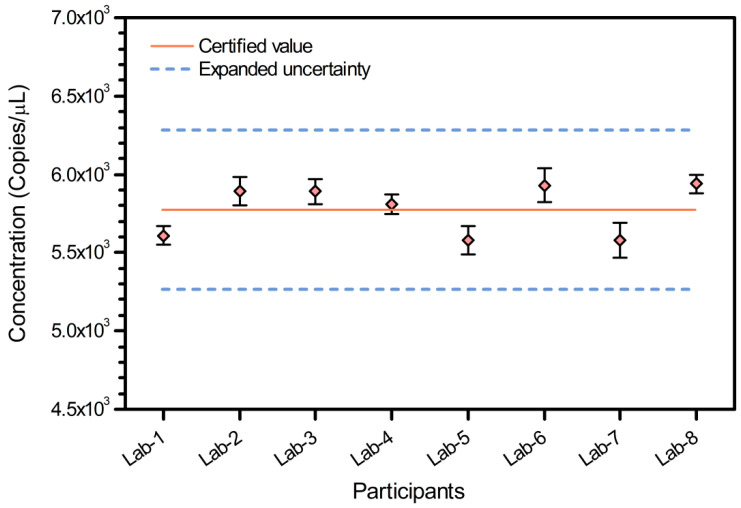
Results for dPCR quantification of the *IL2* gene copy number by eight independent participating laboratories. Solid line, average of participant results (♦); dotted lines, expanded uncertainty of the certified values of the copy number concentration with a 95% level of confidence.

To validate our results through independent third parties, eight different laboratories each determined the *IL2* copy number for two randomly selected duck CRM samples using four technical replicates per sample in dPCR. Thus, each participating external lab returned eight dPCR results (Supplementary Table S6, Figure 3), and the copy numbers were estimated and analyzed according to ISO GUIDE 35:2017. The statistical analysis indicated that the datasets followed a normal distribution, and none of the datasets contained points outside of the uncertainty range for a 95% confidence level. Furthermore, no significant differences were found in the mean *IL2* copy numbers from each lab, and thus, the certified mean *IL2* copy number in the duck CRMs was estimated to be 5.78 × 10^3^ copies/μL.

In addition to *IL2*, mitochondrial genes can also serve as quantifiable targets for the amplification of duck gDNA in meat-based food products. Our laboratory optimized a digital PCR method for detection of the mitochondrial gene *cytb*, which revealed a concentration of 2.0 × 10^6^ copies/μL in the CRM samples.

### 3.5. Statistical Estimation of Uncertainty

The uncertainty of the CRMs for the *IL2* gene in these duck CRMs was estimated from the contributions according to ISO Guide 35 and consisted of uncertainty components from characterization ($u_{\mathrm{char}}$), potential between-unit heterogeneity ($u_{\mathrm{bb}}$), and potential instability during long-term ($u_{\mathrm{its}}$) and short-term storage ($u_{\mathrm{sts}}$).

The relative standard uncertainty of characterization ($u_{\mathrm{char},\mathrm{rel}}$) is estimated by Equation (1). The result is 0.012.

(1) $$u_{\mathrm{char},\mathrm{rel}}=\sqrt{u^{2}{}_{A,\mathrm{rel}}+u^{2}{}_{B,\mathrm{rel}}}$$

where $u_{A,\mathrm{rel}}$ is type A uncertainty (random error) and $u^{2}{}_{B,\mathrm{rel}}$ is type 2 uncertainty (systematic error).

The relative standard uncertainty of potential between-unit heterogeneity ($u_{\mathrm{bb},\mathrm{rel}}$) is 0.0095, estimated as described in Table 1.

The relative standard uncertainty of the potential degradation during transport ($u_{\mathrm{sts},\;\mathrm{rel}}$), and long-term storage ($u_{\mathrm{its},\;\mathrm{rel}}$) is estimated by Equations (2) and (3). The results are 0.035 and 0.020, respectively.

(2) $$u_{\mathrm{sts}}=s\left(\beta_{1}\right)\times X/\bar{x}$$

where $s\left(\beta_{1}\right)$ is the standard deviation of all results of the transport time stability study (Supplementary Table S4), X is the chosen transport time (14 days at 25 °C), and $\bar{x}$ is the mean value.

(3) $$u_{\mathrm{its}}=s\left(\beta_{1}\right)\times X/\bar{x}$$

where $s\left(\beta_{1}\right)$ is the standard deviation of all the results of the long-time stability study (Table S5), X is the chosen transport time (six months at −20 °C), and $\bar{x}$ is the mean value.

The expanded relative uncertainty ($U_{\mathrm{CRM},\mathrm{rel}}$) for the *IL2* gene in these duck CRMs was estimated by Equation (4), and the expanded uncertainty of the copy number concentration ($U_{\mathrm{CRM}}$) was estimated to be 0.51 × 103 (coverage factor *k* = 2, approximate 95% confidence interval).

(4) $$U_{\mathrm{CRM},\mathrm{rel}}=k\times \sqrt{u^{2}{}_{\mathrm{char},\mathrm{rel}}+u^{2}{}_{\mathrm{bb},\mathrm{rel}}+u^{2}{}_{\mathrm{its},\mathrm{rel}}+u_{\mathrm{sts},\mathrm{rel}}^{2}}$$

where $u_{\mathrm{char}}$ is the measurement of the certified value, $u_{\mathrm{bb}}$ is the potential between-unit heterogeneity, $u_{\mathrm{its}}$ and $u_{\mathrm{sts}}$ is the uncertainties in the long-term and short-term stability.

### 3.6. Results of Assay Kit Evaluations

**Figure 4:**
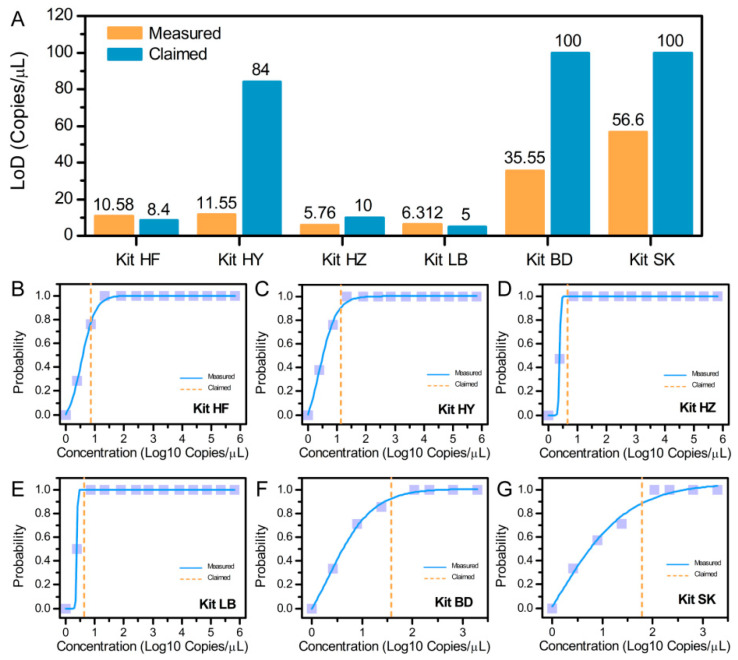
Evaluation of LODs of commercial assays. (**A**) Comparison of LODs claimed by the kit manufacturer using six duck DNA detection kits with the LODs determined with the CRMs. (**B**–**G**) Probit regression analysis of the six assays for the detection of duck *IL2* (SPSS 16.0). The probit versus *IL2* concentration ((**B**–**E**) mitochondrial gene, (**F**,**G**) nuclear gene) was obtained from 21 replicates of serial dilutions and an additional 10 replicates of a blank sample.

We then investigated whether commercially available kits were sufficiently sensitive to be used for extraction of duck gDNA for comparison with CRMs characterized here. For this purpose, we used the externally validated CRM samples and accompanying data to evaluate the LODs of six diagnostic assays (Figure 4). The lot numbers and LODs claimed by each commercial diagnostic assay are listed in Supplementary Table S2. Then, using CRM samples, we tested each of these six kits and compared the results of the LOD determination using a probit regression analysis of 95% hit rates with the LOD in the manufacturer’s instructions for each kit. The results showed that all of the assays met or exceeded their claimed sensitivities and are reliable for detection of *IL2* in gDNA extracted from duck meat. Moreover, the results also show that these duck gDNA CRMs are suitable for the assessment of commercial kits and allow for comparable LOD studies of various detection methodologies.

## 4. Discussion

In recent years, DNA detection methods have been an essential technique for molecular diagnosis. RM and CRM are the quality assurance of testing data, which can evaluate the measurement method and monitor the measurement process. DNA CRMs originated from the detection of genetically modified ingredients because quantitative testing is needed in this field [15]. Subsequently, a large number of molecular diagnostic standards have been developed in the field of medical testing. The international metrology field also pays great attention to DNA measurement, especially traceability to SI units [28]. As an absolute quantitative method, the digital PCR method is currently a potential primary reference measurement method for DNA target measurement. As the carrier of the measurement value, the reference material plays a vital role in the measurement traceability and the guarantee of the data quality of the measurement results.

In this study, we chose the nuclear gene *IL2* as the target gene. Compared with mitochondrial genes, nuclear genes are single-copy genes, genetically stable, and there is no difference between different tissues [6], so it is easy to perform absolute quantification. We fully optimized the *IL2* gene method on qPCR and dPCR platforms. Although the results of this study characterizing a CRM for duck meat are primarily based on dPCR, standard laboratory testing typically relies on qPCR-based analysis. Therefore, it was also necessary to investigate whether the gDNA samples contained inhibitors that could interfere with the qPCR reaction. We first performed six serial dilutions of each gDNA sample, ranging from 100 ng/μL to 0.02 ng/μL, and generated a standard curve of Ct values by qPCR. The linear regression equation between Ct (y) and log10 starting copy number (X) was Y = −3.415X + 42.632. The slope of the curve was −3.415, indicating a 96.3% amplification efficiency and R^2^ = 0.998. The linear regression analysis thus confirmed that the duck gDNA samples were suitably pure for qPCR and met the performance criteria defined by the European Network of GMO Laboratories (ENGL). We next determined that the appropriate linear range for the copy number for subsequent dPCR analysis was between 20 and 14,000 copies per reaction to obtain a good correlation (i.e., R^2^ = 1) with the expected value. Digital PCR commonly uses 20 μL reaction volumes containing 2 μL of the DNA template. The results of the dPCR showed that the *IL2* copy number in these duck CRM samples averaged 5780 copies per microliter, within the linear range, which suggested that 2 μL of the gDNA template was appropriate for analysis. We then determined the standard uncertainty of heterogeneity among technical replicates of each sample to account for variation between samples. The relative uncertainty for the *IL2* copy number was 0.0095, which corresponded with a 95% confidence level. Taken together, these results show that the samples were sufficiently homogenous for accurate evaluation as a standard CRM.

The CRM has two values to meet the detection needs of different targets. The certified value is expressed as (5.78 ± 0.51) × 10^3^ copies/μL with extended uncertainty (a coverage factor *k* = 2) based on the quantification of *IL2* with a collaborative characterization of eight participants. An information value is 2.0 × 10^6^ copies/μL based on the quantification of a mitochondrial gene. Our laboratory determined this value as an informative supplementary value for the quantification of duck meat, which may be of value to users of the CRMs. However, the replication data are currently insufficient to assess the uncertainty associated with this value rigorously.

Lots of novel detection methods of meat adulteration have been developed [29,30], while the literature on reference materials is rarely reported. Some commercial companies have developed RM, but not CRM. Therefore, it is urgent to develop animal DNA standard materials according to the ISO standard system. The identification of meat adulteration is an important part of the field of food safety. DNA-based testing is almost qualitative, and it needs to be further developed into quantitative testing. At the same time, CRM should be developed, international proficiency testing should be organized, and assay kit evaluations should be carried out to ensure accurate and consistent measurement results in the industry.

## 5. Conclusions

Here, we developed gDNA CRMs (GBW(E) 091060) certified by China’s State Administration for Market Regulation for the analysis of duck adulteration in meat products. Eight independent diagnostic laboratories contributed to the validation and certification of these duck gDNA CRMs by digital PCR, confirming a mean of 5.78 ± 0.51 × 10^3^ copies/μL for the *IL2* gene. Homogeneity and stability testing demonstrated that the CRMs were homogenous and stable for at least 6 months at −20 °C storage and for 14 days in cold chain delivery conditions. This batch of duck gDNA CRMs can serve as an essential tool for method validation and proficiency testing in the analysis of duck content in meat products. This study also provides a technical basis for development of other animal-derived reference materials.

## Figures and Tables

**Table 1:** Results of the homogeneity analysis.

Parameter	Copy Number/Unit
Mean	5.76 × 10^3^
Q_1_	3.59 × 10^5^
V_1_	14
S_1_^2^	2.57 × 10^4^
Q_2_	5.02 × 10^4^
V_2_	30
S_2_^2^	1.67 × 10^4^
F	1.53
F_0.05(14, 30)_	2.04
Conclusion	F < F_0.05(14,__30)_
*µ_bbrel_*	0.0095

## Data Availability

Data are available upon request.

## Supplementary Materials

The following are available online at www.mdpi.com/article/10.3390/foods10081890/s1, Table S1: Laboratories participating in the characterization of the *IL2* gene from duck genomic DNA. Table S2: List of commercial assays assessed by the *IL2* certified reference standard. Table S3: Raw data of the homogeneity analysis. Table S4: Results of the short-term stability study. Table S5: Results of the long-term stability study. Table S6: Results of independent laboratory validation.

## References

[1] Köppel R., Daniels M., Felderer N., Brünen-Nieweler C. (2013). Multiplex real-time PCR for the detection and quantification of DNA from duck, goose, chicken, turkey and pork. Eur. Food Res. Technol..

[2] Burns M., Wiseman G., Knight A., Bramley P., Foster L., Rollinson S., Damant A., Primrose S. (2015). Measurement issues associated with quantitative molecular biology analysis of complex food matrices for the detection of food fraud. Analyst.

[3] Kumar A., Kumar R.R., Sharma B.D., Gokulakrishnan P., Mendiratta S.K., Sharma D. (2013). Identification of Species Origin of Meat and Meat Products on the DNA Basis: A Review. Crit. Rev. Food Sci. Nutr..

[4] Böhme K., Calo-Mata P., Barros-Velázquez J., Ortea I. (2019). Review of Recent DNA-Based Methods for Main Food-Authentication Topics. J. Agric. Food Chem..

[5] Kitpipit T., Sittichan K., Thanakiatkrai P. (2013). Are these food products fraudulent? Rapid and novel triplex-direct PCR assay for meat identification. Forensic Sci. Int. Genet. Suppl. Ser..

[6] Li T., Wang J., Wang Z., Qiao L., Liu R., Li S., Chen A. (2021). Quantitative determination of mutton adulteration with single-copy nuclear genes by real-time PCR. Food Chem..

[7] Xu R., Wei S., Zhou G., Ren J., Liu Z., Tang S., Cheung P.C., Wu X. (2018). Multiplex TaqMan locked nucleic acid real-time PCR for the differential identification of various meat and meat products. Meat Sci..

[8] Floren C., Wiedemann I., Brenig B., Schütz E., Beck J. (2015). Species identification and quantification in meat and meat products using droplet digital PCR (ddPCR). Food Chem..

[9] Lin L., Zheng Y., Huang H., Zhuang F., Chen H., Zha G., Yang P., Wang Z., Kong M., Wei H. (2021). A visual method to detect meat adulteration by recombinase polymerase amplification combined with lateral flow dipstick. Food Chem..

[10] Cao Y., Zheng K., Jiang J., Wu J., Shi F., Song X., Jiang Y. (2018). A novel method to detect meat adulteration by recombinase polymerase amplification and SYBR green I. Food Chem..

[11] Wang J., Wan Y., Chen G., Liang H., Ding S., Shang K., Li M., Dong J., Du F., Cui X. (2019). Colorimetric Detection of Horse Meat Based on Loop-Mediated Isothermal Amplification (LAMP). Food Anal. Methods.

[12] Hossain M.A.M., Ali E., Hamid S.B.A., Asing, Mustafa S., Desa M.N.M., Zaidul I.S.M. (2016). Double Gene Targeting Multiplex Polymerase Chain Reaction–Restriction Fragment Length Polymorphism Assay Discriminates Beef, Buffalo, and Pork Substitution in Frankfurter Products. J. Agric. Food Chem..

[13] López-Oceja A., Nuñez C., Baeta M., Gamarra D., de Pancorbo M. (2017). Species identification in meat products: A new screening method based on high resolution melting analysis of cyt b gene. Food Chem..

[14] ISO/TC334 (2015). Reference Materials—Selected Terms and Definitions.

[15] Yang Y., Li L., Yang H., Li X., Zhang X., Xu J., Zhang D., Jin W., Yang L. (2018). Development of Certified Matrix-Based Reference Material as a Calibrator for Genetically Modified Rice G6H1 Analysis. J. Agric. Food Chem..

[16] Li J., Zhang L., Li L., Li X., Zhang X., Zhai S., Gao H., Li Y., Wu G., Wu Y. (2020). Development of Genomic DNA Certified Reference Materials for Genetically Modified Rice Kefeng 6. ACS Omega.

[17] Li J., Li L., Zhang L., Zhang X., Li X., Zhai S., Gao H., Li Y., Wu G., Wu Y. (2020). Development of a certified genomic DNA reference material for detection and quantification of genetically modified rice KMD. Anal. Bioanal. Chem..

[18] Xu J., Qu S., Sun N., Zhang W., Zhang J., Song Q., Lin M., Gao W., Zheng Q., Han M. (2021). Construction of a reference material panel for detecting KRAS/NRAS/EGFR/BRAF/MET mutations in plasma ctDNA. J. Clin. Pathol..

[19] Dong L., Wang X., Wang S., Du M., Niu C., Yang J., Li L., Zhang G., Fu B., Gao Y. (2020). Interlaboratory assessment of droplet digital PCR for quantification of BRAF V600E mutation using a novel DNA reference material. Talanta.

[20] He H.-J., Das B., Cleveland M.H., Chen L., Camalier C.E., Liu L.-C., Norman K.L., Fellowes A.P., McEvoy C.R., Lund S.P. (2019). Development and interlaboratory evaluation of a NIST Reference Material RM 8366 for EGFR and MET gene copy number measurements. Clin. Chem. Lab. Med..

[21] Vallejo C.V., Tere C.P., Calderon M.N., Arias M.M., Leguizamon J.E. (2021). Development of a genomic DNA reference material for Salmonella enteritidis detection using polymerase chain reaction. Mol. Cell. Probes.

[22] Børsting C., Mas C.T., Morling N. (2011). SNP typing of the reference materials SRM 2391b 1–10, K562, XY1, XX74, and 007 with the SNPforID multiplex. Forensic Sci. Int. Genet..

[23] Steffen C.R., Kiesler K.M., Borsuk L.A., Vallone P.M. (2017). Beyond the STRs: A comprehensive view of current forensic DNA markers characterized in the PCR-based DNA profiling standard SRM 2391D. Forensic Sci. Int. Genet. Suppl. Ser..

[24] Cheng X., He W., Huang M. (2013). Establishment and Application of Fluorescent Real-Time PCR for the Detection of Duck Meat in Foods. Food Sci..

[25] SAC/TC387 (2019). Identification of Animal Ingredients from Common Livestock and Poultry Real-Time PCR.

[26] Tholen D.W., Kallner A., Kennedy J.W., Krouver J.S., Meier K. (2004). Evaluation of Precision Performance of Quantitative Measurement Methods; Approved Guideline—Second Edition. https://yeec.com/uploadimages1/forum/2013-2/201321114424796191.pdf.

[27] ISO/Guide 35:2017(en) (2017). Reference Material-Guidance for Characterization and Assessment of Homogeneity and Stability. https://www.iso.org/obp/ui/#iso:std:iso:guide:35:ed-4:v1:en.

[28] Dong L., Meng Y., Wang J., Liu Y. (2014). Evaluation of droplet digital PCR for characterizing plasmid reference material used for quantifying ammonia oxidizers and denitrifiers. Anal. Bioanal. Chem..

[29] Kim M.-J., Lee Y.-M., Suh S.-M., Kim H.-Y. (2020). Species Identification of Red Deer (*Cervus elaphus*), Roe Deer (*Capreolus capreolus*), and Water Deer (*Hydropotes inermis*) Using Capillary Electrophoresis-Based Multiplex PCR. Foods.

[30] Seddaoui N., Amine A. (2021). Smartphone-based competitive immunoassay for quantitative on-site detection of meat adulteration. Talanta.

