# Supplementary material for: Development of a Duck Genomic Reference Material by Digital PCR Platforms for the Detection of Meat Adulteration
Source: Foods. 2021 Aug 15;10(8):1890. doi: 10.3390/foods10081890 (PMC8394454; doi:10.3390/foods10081890)
Supplement: Supplementary file 1 [file foods-10-01890-s001.zip › foods-1318000-supplementary.pdf]

foods

Development of a Duck Genomic Reference Material by Digital PCR Platforms for the Detection of Meat Adulteration

Xiaoyun Chen [1], Yi Ji [1], Kai Li [2], Xiaofu Wang [1], Cheng Peng [1], Xiaoli Xu [1], Xinwu Pei [2], Junfeng Xu [1,*] and Liang Li [2,*]

[1] State Key Laboratory for Managing Biotic and Chemical Threats to the Quality and Safety of Agro-Products, Zhejiang Academy of Agricultural Sciences, Hangzhou 310021, China; xiaoyunchen_2016@163.com (X.C.); jymemory12138@163.com (Y.J.); yywxf1981@163.com (X.W.); pc_phm@163.com (C.P.); xuxiaoli@zju.edu.cn (X.X.)

[2] Biotechnology Research Institute, Chinese Academy of Agricultural Sciences, Beijing 100081, China; likaij@163.com (K.L.); peixinwu@caas.cn (X.P.)

* Correspondence: njjfxu@163.com (J.X.); liliang@caas.cn (L.L.)

Table S1. Laboratories participating in the characterization of the *IL2* gene from duck genomic DNA.

No.	Laboratories	Platform
1	Hangzhou Centre for Inspection and Testing for Quality and Safety of Agricultural and Processed Products, Ministry of Agriculture and Rural Affairs, P. R. China	BioRad QX200
2	Supervision and Test Center for Environmental Safety of Plant and Plant Utilized Microorganisms (Beijing), Ministry of Agriculture and Rural Affairs, P. R. China	BioRad QX200
3	Inspection and Testing Center for Quality of Cereals and Their Products (Harbin), Ministry of Agriculture and Rural Affairs, P. R. China	QuantStudio 3D
4	Zhejiang Academy of Science and Technology for inspection and quarantine	BioRad QX200
5	Environmental Safety Supervision and Inspection Center for Genetically Modified Plants in Hangzhou, Ministry of Agriculture and Rural Affairs	BioRad QX200
6	Supervision and Test Center for Environmental Safety of Plant and Plant Utilized Microorganisms, (Guangzhou) Ministry of Agriculture and Rural Affairs, P. R. China	BioRad QX200
7	The Supervision Inspection & Testing Center of GMO Product Ministry of Agriculture and Rural Affairs (Tianjin)	BioRad QX100
8	Shanghai Supervision Inspection Center Plant Ecology And Environment, Ministry of Agriculture and Rural Affairs	BioRad QX200

Table S2. List of commercial assays assessed by *IL2* certified reference standard.

No.	Product Name	Lot	Target Gene	Claimed LOD
1	Kit HF	48t	Mitochondrial gene	8.4
2	Kit HY	0221052M	Mitochondrial gene	84
3	Kit HZ	HZW0839	Mitochondrial gene	10-100
4	Kit LB	SYA292	Mitochondrial gene	5-10
5	Kit BD	MT-D0503	Nuclear gene	100
6	Kit SK	CHN-D-Kit-18	Nuclear gene	100

Table S3. Raw data of the homogeneity analysis.

Bottles	IL2 (copies/μL)		
	**Rep1**	**Rep2**	**Rep3**
1	5.62E+03	5.82E+03	5.84E+03
2	5.71E+03	5.66E+03	5.76E+03
3	5.85E+03	5.41E+03	5.78E+03
4	5.79E+03	5.80E+03	5.75E+03
5	5.62E+03	5.83E+03	5.80E+03
6	5.90E+03	6.00E+03	5.93E+03
7	5.83E+03	5.55E+03	5.75E+03
8	5.80E+03	5.85E+03	5.93E+03
9	5.64E+03	5.61E+03	5.96E+03
10	5.70E+03	5.76E+03	5.72E+03
11	5.72E+03	5.77E+03	5.82E+03
12	5.75E+03	5.70E+03	5.52E+03
13	5.55E+03	5.66E+03	5.57E+03
14	5.87E+03	6.08E+03	5.78E+03
15	5.99E+03	5.54E+03	5.87E+03

Table S4. Results of the short-term stability study.

date/day	4 °C	25 °C	60 °C
	**Mean Copies**	**Mean Copies**	**Mean Copies**
20190328/0<br>0 day	5.63E+03	5.63E+03	5.63E+03
20190329/1	5.75E+03	5.59E+03	5.60E+03
20190331/3	5.51E+03	5.28E+03	3.66E+03
20190404/7	5.69E+03	5.63E+03	0.63E+03
20190411/14	5.93E+03	5.97E+03	/
mean	5.70E+03	5.62E+03	/
β_1	19.50	18.10	/
$S(\beta_1)$	8.17	10.32	/
$t_{0.95,n-2}$	3.18	3.18	/
$t_{0.95,n-2}$* $S(\beta_1)$	25.99	32.82	/
conclusion	stable	stable	instable

Table S5. Results of the long-term stability study.

Month (Date)	−20 °C
	**Mean Copy Number**
0（20190328）	5.63E+03
1（20190428）	5.44E+03
2（20190528）	5.51E+03
4（20190728）	5.60E+03
6（20190928）	5.58E+03
mean	5.55E+03
β1	6.83
$S(\beta_1)$	17.87
$t_{0.95,n-2}$	3.18
$t_{0.95,n-2}$* $S(\beta_1)$	56.83
conclusion	stable

Table S6. Results of independent laboratory validation.

Lab	Copy Number (×10^3 copies/μL)									
	**Rep1**	**Rep2**	**Rep3**	**Rep4**	**Rep5**	**Rep6**	**Rep7**	**Rep8**	**Mean**	**SD**
1	5.61	5.74	5.58	5.66	5.58	5.55	5.59	5.65	5.61	0.06
2	5.89	5.79	5.71	5.89	5.67	5.83	5.70	5.84	5.89	0.09
3	5.89	5.81	5.92	6.03	5.80	6.00	5.82	5.88	5.89	0.08
4	5.81	5.97	5.81	5.87	5.82	5.88	5.91	5.91	5.81	0.06
5	5.58	5.68	5.59	5.71	5.77	5.78	5.80	5.71	5.58	0.09
6	5.93	5.71	5.60	5.67	5.71	5.85	5.73	5.87	5.93	0.11
7	5.58	5.83	5.63	5.84	5.80	5.72	5.59	5.71	5.58	0.11
8	5.94	5.93	5.92	5.80	5.88	5.96	5.82	5.93	5.94	0.06
Mean						5.78				
SD						0.10				
RSD (%)						1.64				
